# Metabolomic Revelations into the Dynamic Transformations Across Various Developmental Stages of *Coprinus comatus* Through UHPLC-Q-Orbitrap-HRMS Analysis

**DOI:** 10.3390/metabo15110703

**Published:** 2025-10-29

**Authors:** Yu Wang, Guangsheng Ding, Peng Xu, Yun Cheng, Xuan Liang, Chunying Wu, Zhi Yang, Yatuan Ma

**Affiliations:** 1 College of Chemistry & Pharmacy, Northwest A&F University, 22 Xiong Road, Yangling 712100, China; 2 Analysis Center of Agrobiology and Environmental Sciences, Zhejiang University, Hangzhou 310058, China

**Keywords:** *Coprinus comatus*, autolysis, UHPLC-Q-Orbitrap-HRMS, metabolites, KEGG

## Abstract

**Background**: Dietary supplements and functional foods derived from mushrooms have gained increasing popularity. Among these, *Coprinus comatus* stands out due to its excellent flavor and high nutritional value. However, its susceptibility to autolysis and short shelf life significantly limits its utilization. Although a few studies have attempted to elucidate the autolysis mechanism of *C. comatus*, only few research has been conducted on the detailed metabolic changes occurring during its growth cycle. **Objectives**: By conducting a dynamic metabolic profiling analysis of *C. comatus* metabolites across different developmental stages and tissue parts, this study aims to elucidate the variations in its metabolic composition. **Methods**: In this study, fruiting bodies of *C. comatus* were cultivated and collected at four distinct developmental stages. These samples were further divided into cap and gills (CG) and stipe (ST) tissues. Subsequently, UHPLC-Q-Orbitrap was employed for non-targeted dynamic metabolomics analysis of *C. comatus* samples. The identification of analytes was performed using Compound Discovery 3.3. Then, differential accumulated metabolites (DAMs) between CG and ST at the same stage and CG or ST between adjacent stages were identified. Furthermore, Kyoto Encyclopedia of Genes and Genomes (KEGG) pathway analysis was conducted to identify potential contributors to the observed metabolic changes. In addition, the 2,2-diphenyl-1-picrylhydrazyl (DPPH) free radical scavenging activity of samples was determined. **Results**: A total of 490 metabolites were annotated, and the most abundant metabolite groups were lipids, alkaloids, amino acids and their derivatives. It revealed that the metabolites of the ST remained relatively stable across the four growth stages, whereas autolysis induced significant alterations in the metabolites of the CG. KEGG pathway analysis indicated that these changes were primarily linked to lipid and amino acid biosynthesis and metabolic pathways. Furthermore, DPPH assays demonstrated a significant increase in the free radical scavenging activity of CG following autolysis. **Conclusions**: The metabolites of *C. comatus* exhibit dynamic variations across different growth stages and tissue locations. The significant morphological changes in CG induced by autolysis are mirrored by corresponding alterations in its metabolic profile. The enhanced DPPH free radical scavenging activity observed in the autolyzed samples, along with the distribution patterns of bioactive components, provides valuable insights for the efficient utilization of *C. comatus*.

## 1. Introduction

*Coprinus comatus* (O. F. Müll.) Pers. belongs to the genus *Coprinus*, commonly referred to as the shaggy ink cap, lawyer’s wig mushroom or chicken drumstick mushroom. As an edible mushroom, *C. comatus* not only has delicious flavor but it is also rich in carbohydrates, protein, fiber, and phenolics, with low fat content, making it a promising dietary supplement [1]. As a medicinal mushroom, it has been reported to exhibit various pharmacological activities, including antioxidant, hepatoprotective, antidiabetic, antifungal, neuroprotective, insecticidal, anticancer, and analgesic effects [2,3,4,5,6,7,8]. Additionally, *C. comatus* demonstrates significant potential in the areas of functional enzyme development and soil remediation [9,10,11,12]. However, autolysis, a natural self-degradation process, poses the primary challenge to its development and utilization. Once mature, the fruiting bodies will undergo autolysis within a short period of time. Even if harvested during the immature stage, storage at room temperature will result in rapid browning and autolysis, rendering them unsuitable for consumption [13,14]. Conducting a systipeatic metabolic analysis of metabolites in the fruiting bodies at different growth stages is essential to characterize changes in these components and provide a scientific basis for the rational utilization of *C. comatus*.

The mechanism involved the autolysis of *C. comatus* remains unclear. Researchers have employed a variety of approaches to uncover the underlying mechanism, including physiological and biochemical, proteomics, transcriptomics, metabolomics, and bioinformatics analyses. Yi Peng et al. reported that during autolysis, the malondialdehyde (MDA) and reactive oxygen species (ROS) levels showed a gradual upward trend. Meanwhile, enzymes associated with the enzymatic antioxidant defense systipe, such as superoxide dismutase (SOD), peroxidase (POD), and catalase (CAT), exhibited an increase in the early phase of autolysis but subsequently decreased in the later phase. Similarly, enzymes related to cell wall metabolism, including chitinase and β-1,3-glucanase, followed the same pattern of increasing initially and then declining [15]. Proteomics analysis conducted by Hailong Yang et al. revealed that the autolysis mechanism is linked to the hydrolysis of the cell wall and energy biosynthesis prior to pileus opening, whereas the accumulation of ROS and the activation of the mitogen-activated protein kinase (MAPK) signaling pathway occur subsequent to pileus opening [14]. Hongbo Guo et al. utilized RNA-seq to evaluate gene expression across four developmental stages of *C. comatus*. The results indicated that the expression levels of β-1,3-glucanases and chitinase involved in starch and sucrose metabolism, ACOX and ACAA1 associated with fatty acid decomposition, as well as peroxidase-encoding genes such as SOD and PRDX5 were key factors contributing to mushroom autolysis [16]. Integration of transcriptomics and metabolomics was employed to elucidate the protective mechanism of allyl isothiocyanate against *C. comatus* autolysis, revealing that the postharvest quality preservation of mushrooms by allyl isothiocyanate is attributed to the regulation of galactose and sphingolipid metabolism [17].

Although previous studies have sought to elucidate the autolysis mechanism of *C. comatus*, research on autolysis from a metabolomics perspective remains insufficient and requires further investigation. In this study, we cultivated and collected fruiting bodies at four distinct developmental stages. Subsequently, UHPLC-Q-Orbitrap was employed for the non-targeted dynamic metabolomics analysis of the *C. comatus*, aiming to clarify the variations in its metabolic composition across different developmental stages and tissue types. In addition, Kyoto Encyclopedia of Genes and Genomes (KEGG) was utilized for pathway analysis to identify potential contributors to metabolic changes. The study offers valuable insights into the dynamic changes in metabolic components throughout the *C. comatus* growth cycle and their potential for targeted utilization.

## 2. Materials and Methods

### 2.1. Coprinus comatus Cultivation and Collection

**Figure 1:**
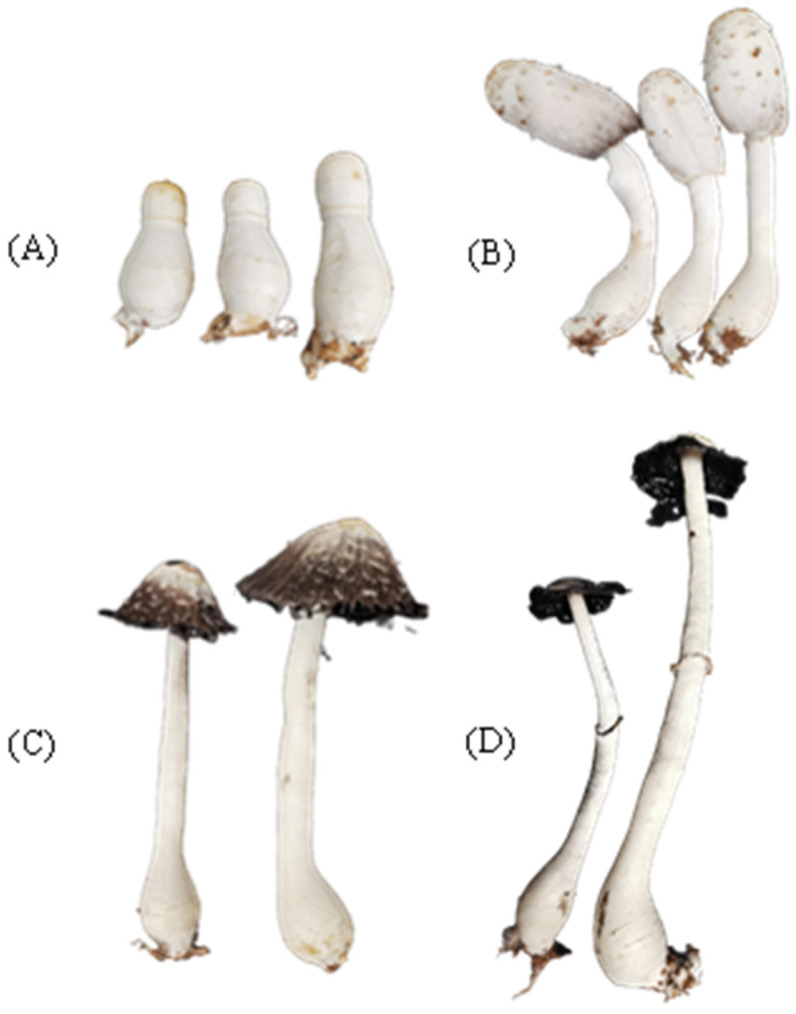
Photographs of the *C. comatus* fruiting body at four different developmental stages: (**A**) the young mushroom stage (Y); (**B**) the maturation stage (M); (**C**) the pre-autolysis stage (PA); and (**D**) the late autolysis stage (LA).

The mycelium packages of *C. comatus* were obtained from Jia Yuan Edible Fungi Planting Farm (Xuzhou, Jiangsu Province, China). The mycelium was thoroughly mixed with sand and soil (*v*/*v*, approximately 1:1:1) and placed in covered a white foam box (40 × 60 × 30 cm, Width, Length, Height) for cultivation. To ensure proper aeration, several small square holes (2 × 2 cm) were made in the lids. The cultivation box was then kept indoors under controlled conditions (July–October, Yangling, Shaanxi; temperature range: 25 ± 10 °C), and the substrate was misted daily to maintain moisture (70 ± 10%). Exposure to astigmatic light was maintained for approximately 12–15 h per day, depending on the natural duration of daylight on each given day. By October, fruiting bodies began to develop. Four distinct developmental stages were selected for analysis: the young mushroom stage (Y), the maturation stage (M), the pre-autolysis stage (PA), and the late autolysis stage (LA) (as shown in Figure 1). These samples were further separated into cap and gills (CG) and stipe (ST) tissues The collected samples were frozen in liquid nitrogen and subsequently stored at −80 °C for metabolomic analysis. The cultivation of *C. comatus* strictly adhered to the guidelines provided by Northwest A&F University.

### 2.2. Metabolomics

#### 2.2.1. Sample Preparation and Extraction

The samples were lyophilized and ground into a fine powder. For metabolomics analysis, 150 mg of each sample was mixed with 1.5 mL of methanol, and the extraction was carried out using ultrasonication (40 kHz, 500 W) at room temperature for 30 min. Following this, the mixtures were centrifuged at 12,000× *g* for 15 min at 4 °C. The supernatant was filtered through a 0.22 μm sintered glass filter, and the resulting filtrate was subjected to UHPLC-HRMS analysis. Each tissue sample was prepared in triplicate. Quality control (QC) samples were prepared by combining 50 μL aliquots from all individual samples, which were then vortex-mixed and carefully transferred into vials for instrumental analysis. The solvent was used as the blank sample. The sample injection sequence was as follows: blank sample, followed by QC-1, QC-2, and QC-3, then Samples 1 through 6, QC-4, Samples 7 through 12, QC-6, and finally, Samples 19 through 24.

#### 2.2.2. UHPLC-HRMS Analysis

The UHPLC–HRMS analysis was carried out using a Vanquish UHPLC system interfaced with a Q-Exactive Focus hybrid quadrupole-Orbitrap mass spectrometer (Thermo Fisher Scientific Inc., Bremen, Germany). Chromatographic separation was performed on a Hypersil GOLD C18 column (1.9 μm, 100 × 2.1 mm; Thermo Fisher Scientific, Waltham, MA, USA), with an injection volume of 3 μL and a column temperature maintained at 30 °C. The mobile phases consisted of solution A (0.1% aqueous formic acid) and solution B (methanol), and the gradient elution program was as follows: 0–15 min, increase B from 10% to 100%; 15–18 min, hold at 100% B; 18–19 min, decrease B from 100% to 10%; 19–23 min, maintain at 10% B, with a constant flow rate of 0.2 mL/min. Mass spectrometric data were acquired in full MS–data-dependent product ion scan (dd-MS2) mode. The mass spectrometer was operated in both positive and negative electrospray ionization (ESI) modes over a mass range of *m/z* 80–1200. The following instrumental parameters were applied: spray voltage, 3.3 kV for negative mode while 3.8 kV for positive mode; sheath gas flow rate, 35 L/min; auxiliary gas flow rate, 5 L/min; capillary temperature, 320 °C; S-lens RF level, 50 V; scan resolutions: 70,000 for full MS and 17,500 for MS/MS; automatic gain control (AGC) targets: 1 × 10^6^ for MS and 2 × 10^5^ for MS/MS; maximum ion injection time for full MS: 100 ms; dynamic exclusion duration: 6 s. The dd-MS2 fragmentation was performed using stepped normalized collision energy (NCE) values of 20%, 40%, and 60%. Instrument control and data acquisition were managed using TraceFinder 5.1 software (Thermo Fisher).

#### 2.2.3. Metabolite Identification and Data Analysis

The non-targeted search and identification of analytes were performed using Compound Discovery 3.3 and Xcalibur™ 4.4 software (Thermo Scientific, Waltham, MA, USA). The analysis was carried out following the software’s built-in workflow “Untargeted Metabolomics with Statistics Detect Unknowns with ID using Online Databases.” The parameters for “Assign Compound Annotations” were set with the following constraints: a mass tolerance of 5 ppm; data sources included mzCloud Search, Predicted Compositions, MassList Search, ChemSpider Search, and Metabolika Search. The minimum peak intensity was set to 3 × 10^6^ for “Detect Compounds”. Other parameters were based on the default settings.

Multivariate statistical analysis was conducted on the acquired metabolomics data. Initially, principal component analysis (PCA) was employed to visualize the overall metabolic differences among samples and to assess the variation across groups [18]. Filtering features were based on QC samples (RSDs < 30%). Sample normalization was performed using the sum method. Data transformation was conducted via logarithmic transformation (base 10), followed by Pareto scaling for data scaling. Subsequently, volcano plots analysis was performed for the differential accumulated metabolites (DAMs) identification across four distinct developmental stages using the following criteria: fold change (FC) ≥ 2 or ≤0.5, and *p*-value < 0.05. The Kyoto Encyclopedia of Genes and Genomes (KEGG) database was used to annotate the potential biochemical pathways associated with these DAMs. Statistical analyses were carried out using the MetaboAnalyst platform (https://www.metaboanalyst.ca/home.xhtml, accessed on 26 September 2025), Rstudio and Origin 2021.

### 2.3. Determination of Total Phenols and DPPH Free Radical Scavenging Activity

For the determination of total phenols and 2,2-diphenyl-1-picrylhydrazyl (DPPH) free radical scavenging activity, 500 mg of CG or ST tissue was extracted with 5 mL of methanol via ultrasonication at room temperature for 30 min. The mixtures were centrifuged at 12,000× *g* for 15 min at 4 °C prior to analysis, and the resulting supernatant was used for subsequent measurements.

The total phenolic content of all samples was determined using the Folin–Ciocalteu method [19]. A standard curve for total phenolic content was constructed using various concentrations of gallic acid, and absorbance was measured at 760 nm. For analysis, 25 μL of each extract or gallic acid solution was placed into a 96-well plate, followed by the addition of Folin–Ciocalteu reagent (25 μL). The mixture was kept in the dark at room temperature for 5 min. Subsequently, 100 μL of 20% Na_2_CO_3_ was added, and the samples were incubated in the dark at room temperature for an additional 30 min. Absorbance was then measured at 760 nm using a spectrophotometer (BioTek Synergy HTX, Agilent, CA, USA). Total phenolic content was expressed as milligrams of gallic acid equivalent per gram of tissue. All analyses were performed in triplicate.

The DPPH radical scavenging activity was assessed using the 2,2-diphenyl-1-picrylhydrazyl (DPPH) assay, as originally described by W. Brand-Williams et al. [20]. In brief, 50 μL of the extract and 50 μL of DPPH radical solution (100 μM in methanol) were added to a 96-well plate, thoroughly mixed, and incubated in the dark at 30 °C for 30 min. The absorbance was then measured at 517 nm. The radical scavenging activity was calculated according to Equation (1):

(1) DPPH scavenging (%) = (A_control_ − A_sample_)/A_control_ × 100%

## 3. Results

### 3.1. Coprinus comatus Cultivation and Collection

The life cycle of *C. comatus* consists of a prolonged spore germination and mycelial growth phase, followed by a relatively short fruiting body development stage. In this experiment, the mycelium grew for more than two months before initiating fruiting body formation, whereas the period from fruiting body formation to autolysis lasted only about two weeks. When the fruiting body reached maturity, the color of both the cap and the gills gradually darkened, and autolysis occurred rapidly within 1–2 days. To investigate the dynamic changes in metabolite composition during fruiting body development, we collected samples at four distinct developmental stages: Y, M, PA, and LA. These samples were further divided into CG and ST tissues. Subsequently, a non-targeted metabolomics analysis was conducted using UHPLC-Q-Orbitrap technology.

### 3.2. Analysis of Metabolite in the Four Stages of the Coprinus comatus Fruiting Body

The analysis of the quality control (QC)-total ion current (TIC) plot (Figure S1A,B) revealed a high degree of overlap in the total ion flow curves of detected metabolites, including both retention time and peak intensity. This indicates that the LC-MS systipe maintained a stable signal when analyzing the same sample at different time points.

**Figure 2:**
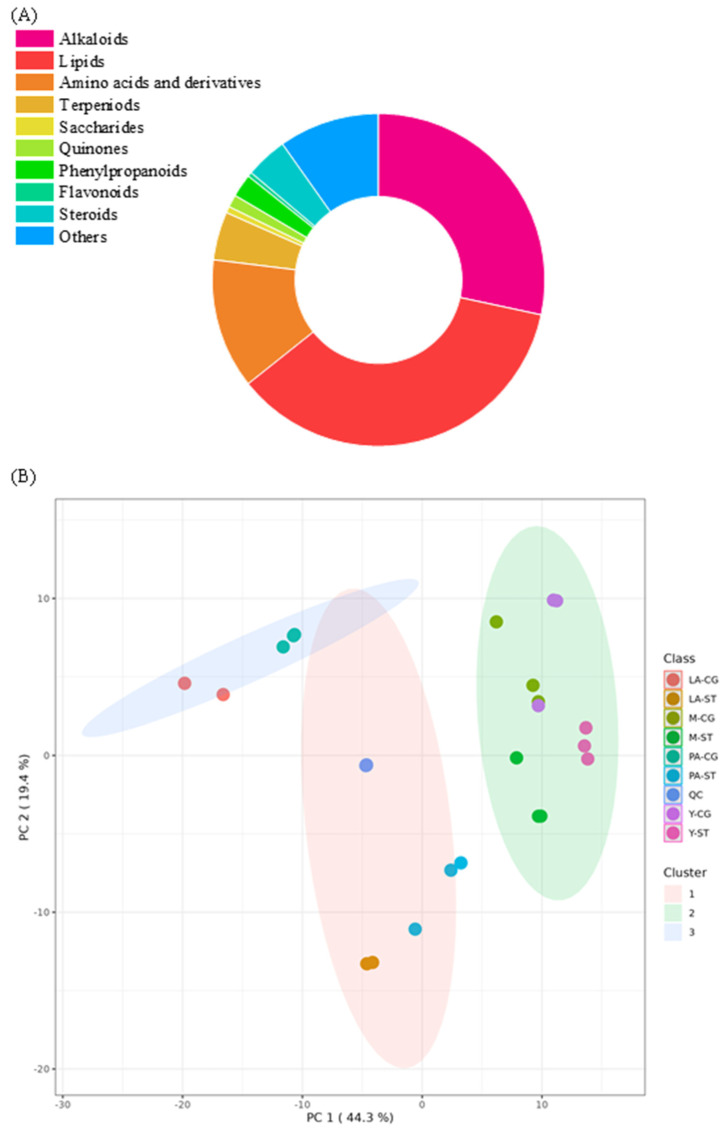
(**A**) Classification of the identified metabolites in the *C. comatus* fruiting body. (**B**) Principal component analysis of Y-CG, Y-ST, M-CG, M-ST, PA-CG, PA-ST, LA-CG, LA-ST, and QC samples.

In the present study, a total of 490 metabolites from the fruiting bodies of *C. comatus* at four developmental stages were annotated, including 337 compounds detected in positive ion mode and 153 in negative ion mode (shown in Table S1) based on both primary and secondary mass spectrometry simultaneously. In addition, ergothioneine was further confirmed by a standard substance. These metabolites were classified into 10 major categories. As shown in Figure 2A, the most abundant metabolite groups were lipids (176), alkaloids (139), amino acids and their derivatives (62), terpenoids (23), and steroids (20). To provide an overview of the metabolic variation among the samples, principal component analysis (PCA) was conducted. As shown in Figure 2B, all quality control (QC) samples were closely clustered, indicating consistent analytical performance (QC samples (n = 6): median RSD = 1.5%; 100.0% of features < 30%). These findings demonstrate the stability of the metabolite monitoring process and confirm the reliability and reproducibility of the acquired data. In addition, three distinct clusters were observed: one comprising CG and ST samples from the Y and M stages, one including CG samples from the PA and LA stages, and another consisting of ST samples from the PA and LA stages. These results suggest that following the initiation of autolysis in *C. comatus* may significant metabolic alterations occurred in the samples.

### 3.3. Analysis of the Metabolite Differences Between CG and ST at the Same Stage and CG or ST Between Adjacent Stages

**Figure 3:**
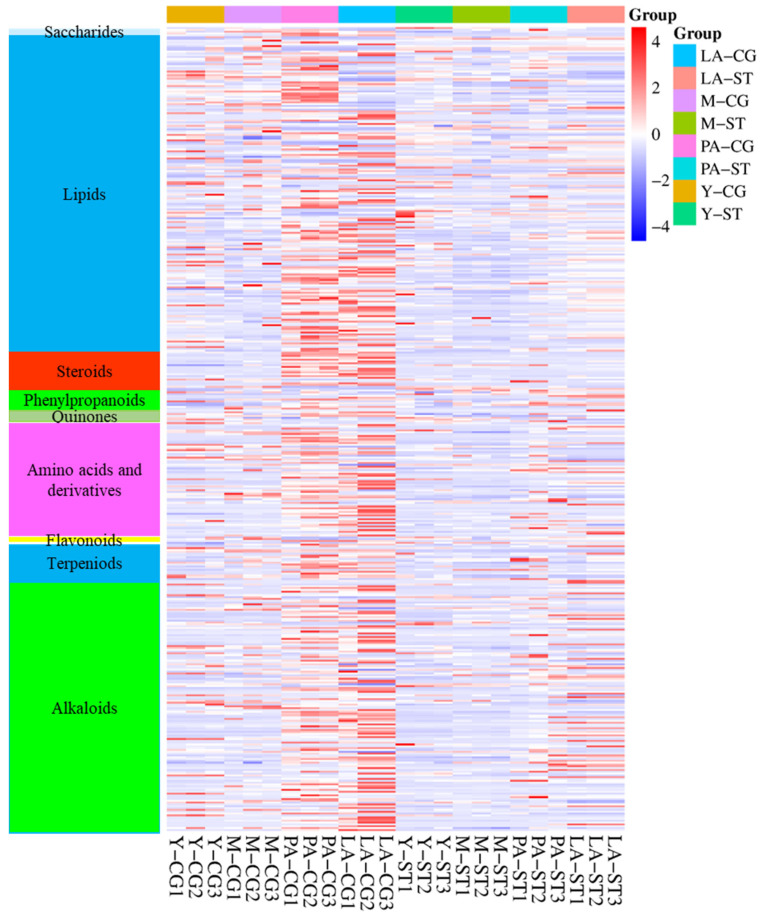
The heatmaps of metabolites in the cap and gills (CG) and stipe (ST) tissues samples.

To investigate metabolic differences between CG and ST at the same developmental stage, as well as within CG or ST across adjacent stages, volcano plot analyses were conducted for the following pairwise comparisons: Y-CG vs. Y-ST, M-CG vs. M-ST, PA-CG vs. PA-ST, LA-CG vs. LA-ST, Y-CG vs. M-CG, M-CG vs. PA-CG, PA-CG vs. LA-CG, Y-ST vs. M-ST, M-ST vs. PA-ST, and PA-ST vs. LA-ST. Combined with a fold change (FC) ≥ 2 or ≤0.5 and a *p*-value < 0.05, these criteria were used to determine significant changes in metabolite levels. Additionally, the false discovery rate (FDR) was calculated using the Benjamini–Hochberg method to further validate the identified DAMs. As shown in Figure S2, the volcano plots illustrate the number of differentially accumulated metabolites (DAMs) across the compared groups. Specifically, 28 DAMs were identified between Y-CG and Y-ST (24 up-regulated and 4 down-regulated), 47 between M-CG and M-ST (34 up-regulated and 13 down-regulated), 185 between PA-CG and PA-ST (145 up-regulated and 40 down-regulated), 227 between LA-CG and LA-ST (153 up-regulated and 74 down-regulated), 39 between Y-CG and M-CG (18 up-regulated and 21 down-regulated), 235 between M-CG and PA-CG (33 up-regulated and 202 down-regulated), 70 between PA-CG and LA-CG (34 up-regulated and 36 down-regulated), 32 between Y-ST and M-ST (6 up-regulated and 26 down-regulated), 4 between M-ST and PA-ST (2 up-regulated and 2 down-regulated), and 12 between PA-ST and LA-ST (4 up-regulated and 8 down-regulated). The classification of DAMs between CG and ST at the same developmental stage, as well as between CG or ST across adjacent stages, was presented in Table 1. Additionally, the metabolite heatmaps for the CG and ST samples across the lifecycle stages were presented in Figure 3. It was evident that the changes in metabolites during autolysis were highly complex.

### 3.4. KEGG Enrichment Analysis of DAMs Between Adjacent CG Samples

From the above results, it can be observed that the metabolites in ST tissues remained relatively stable, whereas the metabolic components in CG tissues underwent significant changes, which were closely associated with spore development, maturation, and the autolysis process. To identify the key metabolic pathways involved, KEGG analysis was performed on the DAMs.

Although 39 DAMs were identified between Y-CG and M-CG, no pathway was significantly enriched in the KEGG pathway enrichment analysis. This may be because spore formation does not induce substantial metabolic alterations, or because metabolites with significant changes have not yet been detected or identified.

**Figure 4:**
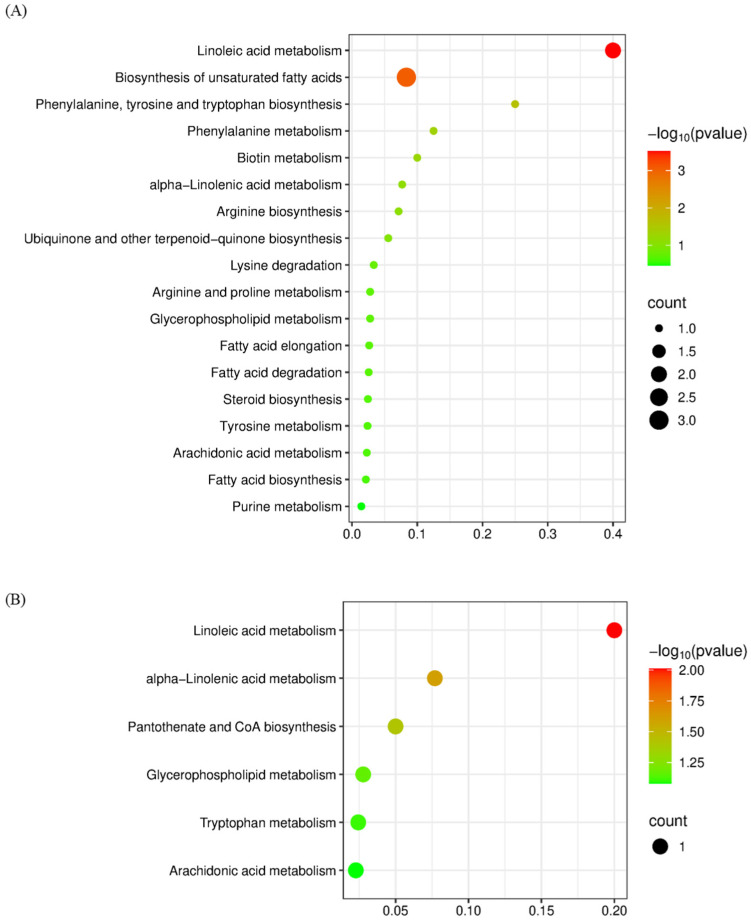
(**A**) KEGG analysis between M-CG and PA-CG; (**B**) KEGG analysis between PA-CG and LA-CG.

For M-CG and PA-CG, 235 DAMs were identified, with 18 enriched pathways, most of which were associated with lipid and amino acid biosynthesis and metabolism (shown in Figure 4A). For PA-CG and LA-CG, the 6 pathways were enriched, including linoleic acid and α-Linolenic acid metabolism, pantothenate and CoA biosynthesis, glycerophospholipid metabolism, tryptophan metabolism, and arachidonic acid metabolism. Therefore, autolysis is a highly regulated and intricate process governed by multiple metabolic pathways.

### 3.5. Determination of Total Phenols and DPPH Free Radical Scavenging Activity

The unique feature of *C. comatus* is that it is edible only when young; the older specimens undergo rapid autolysis, which significantly increases postharvest storage difficulties, restricts logistics capabilities, and adversely affects the preservation of edible mushrooms [13]. However, mature and autolysis can also offer certain benefits in specific contexts. P. Petrovic et al. found that mature puffball fruiting body extracts contained higher concentrations of phenolic compounds, α-tocopherol, and ergosterol compared to those of immature specimens, resulting in enhanced antioxidant activity. Moreover, although autolysis may reduce the antimicrobial potential of puffball extracts, they still exhibit considerable inhibitory activity, including against methicillin-resistant *Staphylococcus aureus* [21]. In this study, the total phenolic contents and DPPH free radical scavenging activity were determined, aiming to identify a potential breakthrough for utilizing mature or autolysis specimens of *C. comatus*. As shown in Table 2, the concentrations of total phenols in the ST part were higher than those in the CG part at the same developmental stage, with the highest concentration observed in PA-ST. Notably, a high total phenol content does not necessarily correlate with high DPPH free radical scavenging activity.

**Figure 5:**
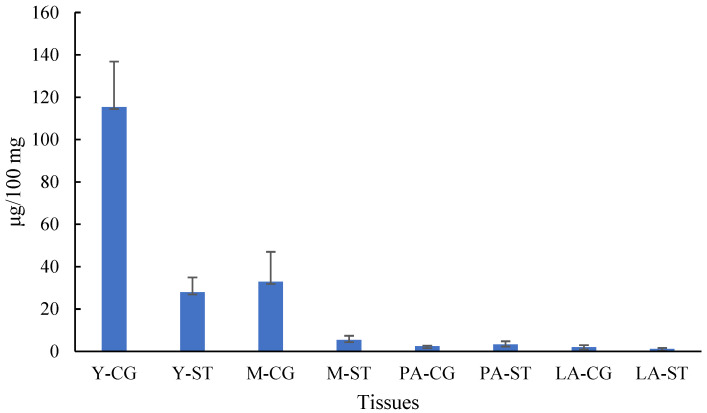
The ergothioneine content in cap and gills (CG) and stipe (ST) samples.

In addition to the total content of various substances and overall biological activity, the differences in the content of certain key monomeric components are also crucial for their effective utilization. Ergothioneine is synthesized exclusively by certain fungi and bacteria, and not by animals or higher plants. Ergothioneine not only exhibits therapeutic potential against various diseases [22,23], but is also widely applied in processed foods and cosmetics [24,25]. Mushrooms, together with several other fungi capable of ergothioneine biosynthesis, represent a major dietary source of this compound for humans [25]. Studies have shown that *C. comatus* contains a significant amount of ergothioneine [26]. Therefore, the ergothioneine content was analyzed at different developmental stages and in various tissues. As illustrated in Figure 5, ergothioneine was predominantly present at the Y and M stages, with its concentration in the CG tissue being notably higher than in the ST tissue. The highest concentration of ergothioneine in CG tissue was observed at the Y stage, exceeding 0.1%.

## 4. Discussion

In the study by Yilong Zou et al., a mixed solvent systipe (methanol/acetonitrile/water = 2:2:1 for extraction and acetonitrile/water = 1:1 for redissolution) was used, and the majority of identified metabolites were also the lipids [17]. Among the identified fatty acids, most are unsaturated, with linoleic acid present in the highest concentration, a finding consistent with the results reported by Nebojša Stilinović et al. [1]. Because the solvent used in this experiment has lower polarity, a higher proportion of lipid components was detected. This finding suggests that the selection of extraction solvent significantly influences metabolite identification.

From a morphological standpoint, the CG and ST tissues exhibited minimal differences at the Y stage. As development progressed to the M stage, browning began to appear in the CG region, resulting in a noticeable color divergence from the ST region. This distinction became increasingly pronounced during autolysis. For the ST region, the most prominent change is the significant increase in length, whereas the color remains virtually unchanged. The alterations in metabolic components are as follows: For samples from the same developmental period but different tissues, the Y stage exhibited the lowest number of DAMs. Upon transitioning into the M stage, spore formation in the CG region triggered an increase in DAMs, and a sharp rise was observed after entering the autolysis stage. In contrast, metabolic profiles in the ST region remained relatively stable from the M to the LA stage, with only 4 and 12 DAMs detected between adjacent stages, respectively. This suggests that the initiation and progression of autolysis drive substantial metabolic alterations specifically within the CG region. The significantly elevated number of DAMs between the M and PA stages in the CG region further corroborates this conclusion. Autolysis significantly affects metabolic components, as the activities of multiple enzymes undergo substantial alterations, resulting in cell structural degradation, protein hydrolysis, and shifts in oxidative stress levels [15,17]. These biochemical transformations directly influenced amino acid, lipid, and steroid profiles. As illustrated in Figure 3, the content changes of these three component classes became upon entry into the PA stage. Transcriptome analysis was conducted by Hongbo Guo et al. on CG tissues at four distinct developmental stages of *C. comatus*: the infant stage (I), mature stage (M), discolored stage (D), and autolysis stage (A) [16]. The first two stages correspond to the Y and M phases, while the latter two correspond to the PA phases, respectively, in this study. Their results indicated that the number of shared genes between stages I and M was higher than that between M and D. Similarly, this study found that the number of DAMs in the CG samples between the M and PA phases was significantly greater than that between the Y and M phases. In addition, the expression levels of β-1,3-glucanases and chitinase were found to be significantly up-regulated, potentially contributing to cell wall degradation. Meanwhile, the expression levels of ACOX and ACAA1, which are involved in fatty acid degradation, were also elevated. Consistently, this study identified 86 lipid DAMs—values higher than those observed in other groups. Proteomic and transcriptomic analyses both indicate that fatty acid biosynthesis and metabolism play crucial roles in this autolytic process [16,27], a finding consistent with the metabolomics results obtained in this study. It demonstrated that the findings from various omics studies were consistent.

ROS are highly reactive oxygen metabolites, such as O_2_^∙−^ and H_2_O_2_, that are generally regarded as toxic by-products of plant metabolism and can induce oxidative damage to macromolecules, including lipids [28]. Superoxide dismutase (SOD), peroxidase (POD), and catalase (CAT) are key antioxidant enzymes in edible fungi, playing a crucial role in the antioxidant defense systipe by protecting membrane integrity during fungal autolysis [29]. The upregulation or downregulation of key enzymes disrupts cellular integrity, thereby inducing severe oxidative stress that promotes the degradation of biomolecules such as proteins and lipids. These alterations are reflected in metabolomic profiles through significant shifts in the levels of lipids, amino acids, steroids, and other metabolites. Studies by Yi Peng et al. and Hailong Yang et al. demonstrated that in *C. comatus*, oxidative stress progressively increased during autolysis. However, the activities of major antioxidant enzymes initially increased and then declined, with the turning point coinciding with severe cell membrane damage, leading to pronounced membrane lipid peroxidation [14,15]. Consequently, for PA and LA samples, the enriched metabolic pathways were predominantly associated with lipid metabolism (shown in Figure 4B). Transcriptomic and proteomic analyses both revealed that autolysis induced alterations in numerous proteins and transcription factors [14,16]. These molecular changes may contribute to an increased accumulation of antioxidant-related components, such as malondialdehyde (MDA) [14,15]. As illustrated in Figure 3, both PA-CG and LA-CG exhibited higher levels of amino acids, lipids, and steroids, which may contribute to enhanced antioxidant activity. In this manner, mature or autolyzed fruiting bodies that are no longer suitable for primary use can serve as valuable raw materials for the development of alternative products, such as antioxidant agents.

## 5. Conclusions

In this study, a non-targeted metabolomics approach was employed to analyze cap and gills (CG) and stipe (ST) samples collected at four distinct time points using UHPLC-Q-Orbitrap-HRMS. The results revealed that autolysis induces significant alterations in metabolic profiles, particularly related with the lipid and amino acid biosynthesis and metabolism. Although maturation and autolysis render *C. comatus* unsuitable for direct consumption, the observed metabolic shifts—such as increased antioxidant activity—suggest potential applications in the development of natural antioxidants. These findings provide valuable insights into the dynamic changes in chemical composition during *C. comatus* fruiting body development and lay the groundwork for the targeted utilization of its bioactive components.

## Figures and Tables

**Table 1:** DAMs between cap and gills (CG) and stipe (ST) tissues at the same stage and between adjacent stages.

Classification	Y-CG/Y-ST		M-CG/M-ST		PA-CG/PA-ST		LA-CG/LA-ST		Y-CG/M-CG		M-CG/PA-CG		PA-CG/LA-CG		Y-ST/M-ST		M-ST/PA-ST		PA-ST/LA-ST	
	Down	Up	Down	Up	Down	Up	Down	Up	Down	Up	Down	Up	Down	Up	Down	Up	Down	Up	Down	Up
Alkaloids		8	7	10	4	35	26	50	9	4	65	7	14	8	14					2
Lipids	1	9		10	17	58	20	44	6	9	74	12	11	18	2	4	2	1	6	1
Amino acids derivatives		4	3	7	3	19	8	24	5	1	30	5	4	4	10	1				1
Terpeniods	1		1		1	8	1	11		1	5	2								
Saccharide	1						1							1						
Quinones						3		3						1						
Flavonoids						1		1					1							
Phenylpropanoids	1		1	1		1	4	2		1			1						1	
Steroids				4	2	14		11	1		14	2	1	1		1			1	
Others		3	1	2	13	6	14	7		2	14	5	4	1				1		
Sum	4	24	13	34	40	145	74	153	21	18	202	33	36	34	26	6	2	2	8	4

**Table 2:** Determination of total phenols and DPPH free radical scavenging activity.

Samples	Y-CG	M-CG	PA-CG	LA-CG	Y-ST	M-ST	PA-ST	LA-ST
Total phenols (mg/g)	9.33 ± 0.08	8.09 ± 0.13	8.12 ± 0.35	4.46 ± 0.47	10.10 ± 0.02	12.48 ± 0.22	13.03 ± 0.17	7.87 ± 0.27
DPPH∙scavenging activity (%)	71.8 ± 2.2	65.0 ± 8.1	88.8 ± 2.4	93.7 ± 0.9	51.2 ± 3.8	52.9 ± 6.9	61.1 ± 8.7	56.8 ± 3.1

## Data Availability

The data has been uploaded on metabolights (MTBLS13133).

## Supplementary Materials

The following supporting information can be downloaded at: https://www.mdpi.com/article/10.3390/metabo15110703/s1, and MTBLS13133. Figure S1: Total ions chromatography (TIC) overlapping map of QC samples in the mass spectrometry results. (A) was detected in the positive ion mode; (B) was detected in the negative ion mode. Figure S2: Analysis of metabolites between CG and ST at the same stage and CG or ST between adjacent stages. Volcano plots. (A), Y-CG vs. Y-ST; (B), M-CG vs. M-ST; (C), PA-CG vs. PA-ST; (D), LA-CG vs. LA-ST; (E), Y-CG vs. M-CG; (F), M-CG vs. PA-CG; (G), PA-CG vs. LA-CG; (H), Y-ST vs. M-ST; (I), M-ST vs. PA-ST; (J), PA-ST vs. LA-ST. Table S1: The annotated metabolites information. Table S2: FDR T-test.

## Abbreviations

The following abbreviations are used in this manuscript:

CG	cap and gills tissues
ST	stipe tissues
DAM	differential accumulated metabolites
KEGG	Kyoto Encyclopedia of Genes and Genomes
MDA	malondialdehyde
ROS	reactive oxygen species
SOD	superoxide dismutase
POD	peroxidase
CAT	catalase
MAPK	mitogen-activated protein kinase
Y	the young mushroom stage
M	the maturation stage
PA	the pre-autolysis stage
LA	the late autolysis stage
QC	quality control
TIC	total ion current
PCA	principal component analysis
dd-MS2	MS–data-dependent product ion scan
ESI	electrospray ionization
AGC	automatic gain control
NCE	normalized collision energy
VIP	variable importance in projection
FC	fold change
DPPH	2,2-diphenyl-1-picrylhydrazyl

## References

[1] Stilinovic N., Capo I., Vukmirovic S., Raskovic A., Tomas A., Popovic M., Sabo A. (2020). Chemical composition, nutritional profile and in vivo antioxidant properties of the cultivated mushroom *Coprinus comatus*. R. Soc. Open Sci..

[2] Zhao H.J., Li H.P., Lai Q.Q., Yang Q.H., Dong Y.H., Liu X.C., Wang W.S., Zhang J.J., Jia L. (2019). Antioxidant and hepatoprotective activities of modified polysaccharides from *Coprinus comatus* in mice with alcohol-induced liver injury. Int. J. Biol. Macromol..

[3] Gao Z., Kong D.Y., Cai W.X., Zhang J.J., Jia L. (2021). Characterization and anti-diabetic nephropathic ability of mycelium polysaccharides from *Coprinus comatus*. Carbohy. Polym..

[4] Cabutaje E.M., Seki K., Kodama M., Arie T., Ueno K., Cruz T.E.E.D., Ishihara A. (2024). Coprinolide, a novel antifungal tricyclic polyketide with a rare furanone-fused chromene skeleton isolated from the mushroom *Coprinus comatus*. J. Pestic. Sci..

[5] Karaman M., Tesanovic K., Novakovic A., Jakovljevic D., Janjusevic L., Sibul F., Pejin B. (2020). *Coprinus comatus* filtrate extract, a novel neuroprotective agent of natural origin. Nat. Prod. Res..

[6] Luo H., Mo M.H., Huang X.W., Li X., Zhang K.Q. (2004). *Coprinus comatus*: A basidiomycete fungus forms novel spiny structures and infects nematode. Mycologia.

[7] Ren J., Shi J.L., Han C.C., Liu Z.Q., Guo J.Y. (2012). Isolation and biological activity of triglycerides of the fermented mushroom of *Coprinus comatus*. BMC Complement. Altern. Med..

[8] Nowakowski P., Markiewicz-Zukowska R., Gromkowska-Kepka K., Naliwajko S.K., Moskwa J., Bielecka J., Grabia M., Borawska M., Socha K. (2021). Mushrooms as potential therapeutic agents in the treatment of cancer: Evaluation of anti-glioma effects of *Coprinus comatus*, *Cantharellus cibarius*, *Lycoperdon perlatum* and *Lactarius deliciosus* extracts. Biomed. Pharmacother..

[9] Wang Y., Zhang B.W., Chen N.J., Wang C., Feng S., Xu H. (2018). Combined bioremediation of soil co-contaminated with cadmium and endosulfan by *Pleurotus eryngii* and *Coprinus comatus*. J. Soils Sediments.

[10] Bao S.Y., Teng Z., Ding S.J. (2013). Heterologous expression and characterization of a novel laccase isoenzyme with dyes decolorization potential from *Coprinus comatus*. Mol. Biol. Rep..

[11] Falandysz J. (2016). Mercury bio-extraction by fungus Coprinus comatus: A possible bioindicator and mycoremediator of polluted soils?. Environ. Sci. Pollut. Res..

[12] Wang J.Y., Liu X.L., Jing Y., Zheng X.Q. (2024). Purification and biochemical characterization of a novel fibrinolytic enzyme from culture supernatant of *Coprinus comatus*. Foods.

[13] Nowakowski P., Naliwajko S.K., Markiewicz-Zukowska R., Borawska M.H., Socha K. (2020). The two faces of *Coprinus comatus*—Functional properties and potential hazards. Phytother. Res..

[14] Yang H.L., Zheng Z.H., Zhou H.B., Qu H., Gao H.Y. (2022). Proteomics reveals the mechanism underlying the autolysis of postharvest *Coprinus comatus* fruiting bodies. J. Agric. Food Chem..

[15] Peng Y., Li T.L., Jiang H.M., Gu Y.F., Chen Q., Yang C.R., Qi W.L., Liu S.Q., Zhang X.P. (2020). Postharvest biochemical characteristics and ultrastructure of *Coprinus comatus*. PeerJ.

[16] Guo H.B., Zhang Z.F., Wang J.Q., Wang S.Y., Yang J.K., Xing X.Y., Qi X.J., Yu X.D. (2022). Transcriptome analysis of genes associated with autolysis of *Coprinus comatus*. Sci. Rep..

[17] Zou Y.L., Zhang Y.R., Zheng E.P., Zhou H.B., Qu H., Yang H.L. (2024). Integration of transcriptomics and metabolomics to reveal the mechanism of allyl isothiocyanate delaying postharvest quality deterioration of *Coprinus comatus*. Postharvest Biol. Technol..

[18] Claassen C., Kuballa J., Rohn S. (2019). Metabolomics-based approach for the discrimination of potato varieties (*Solanum tuberosum*) using UPLC-IMS-QToF. J. Agric. Food Chem..

[19] Prior R.L., Wu X.L., Schaich K. (2005). Standardized methods for the determination of antioxidant capacity and phenolics in foods and dietary supplements. J. Agric. Food Chem..

[20] Brand-Williams W., Cuvelier M.E., Berset C. (1995). Use of a free-radical method to evaluate antioxidant activity. Food Sci. Technol..

[21] Petrovic P., Ivanovic K., Jovanovic A., Simovic M., Milutinovic V., Kozarski M., Petkovic M., Cvetkovic A., Klaus A., Bugarski B. (2019). The impact of puffball autolysis on selected chemical and biological properties: Puffball extracts as potential ingredients of skin-care products. Arch. Biol. Sci..

[22] Cheah I.K., Halliwell B. (2012). Ergothioneine; antioxidant potential, physiological function and role in disease. Biochim. Biophys. Acta Mol. Basis Dis..

[23] Nakamichi N., Tsuzuku S., Shibagaki F. (2022). Ergothioneine and central nervous systipe diseases. Neurochem. Res..

[24] Borodina I., Kenny L.C., McCarthy C.M., Paramasivan K., Pretorius E., Roberts T.J., van der Hoek S.A., Kell D.B. (2020). The biology of ergothioneine, an antioxidant nutraceutical. Nutr. Res. Rev..

[25] Xiong K., Xue S., Guo H., Dai Y., Ji C., Dong L., Zhang S. (2024). Ergothioneine: New functional factor in fermented foods. Crit. Rev. Food Sci. Nutr..

[26] Asahi T., Wu X., Shimoda H., Hisaka S., Harada E., Kanno T., Nakamura Y., Kato Y., Osawa T. (2016). A mushroom-derived amino acid; ergothioneine, is a potential inhibitor of inflammation-related DNA halogenation. Biosci. Biotechnol. Biochem..

[27] Zha L., Chen M., Guo Q., Tong Z., Li Z., Yu C., Yang H., Zhao Y. (2022). Comparative proteomics study on the postharvest senescence of *Volvariella volvacea*. J. Fungi.

[28] Desikan R., Cheung M.K., Clarke A., Golding S., Sagi M., Fluhr R., Rock C., Hancock J., Neill S. (2004). Hydrogen peroxide is a common signal for darkness- and ABA-induced stomatal closure in *Pisum sativum*. Funct. Plant Biol..

[29] Xing Z., Wang Y., Feng Z., Zhao Z., Liu X. (2007). Effect of ^60^Co-irradiation on postharvest quality and selected enzyme activities of *Hypsizygus marmoreus* fruit bodies. J. Agric. Food Chem..

