# Supplementary material for: Metabolomic Revelations into the Dynamic Transformations Across Various Developmental Stages of Coprinus comatus Through UHPLC-Q-Orbitrap-HRMS Analysis
Source: Metabolites. 2025 Oct 29;15(11):703. doi: 10.3390/metabo15110703 (PMC12654644; doi:10.3390/metabo15110703)
Supplement: Supplementary file 1 [file metabolites-15-00703-s001.zip › Supplementary material.pdf]

(A)

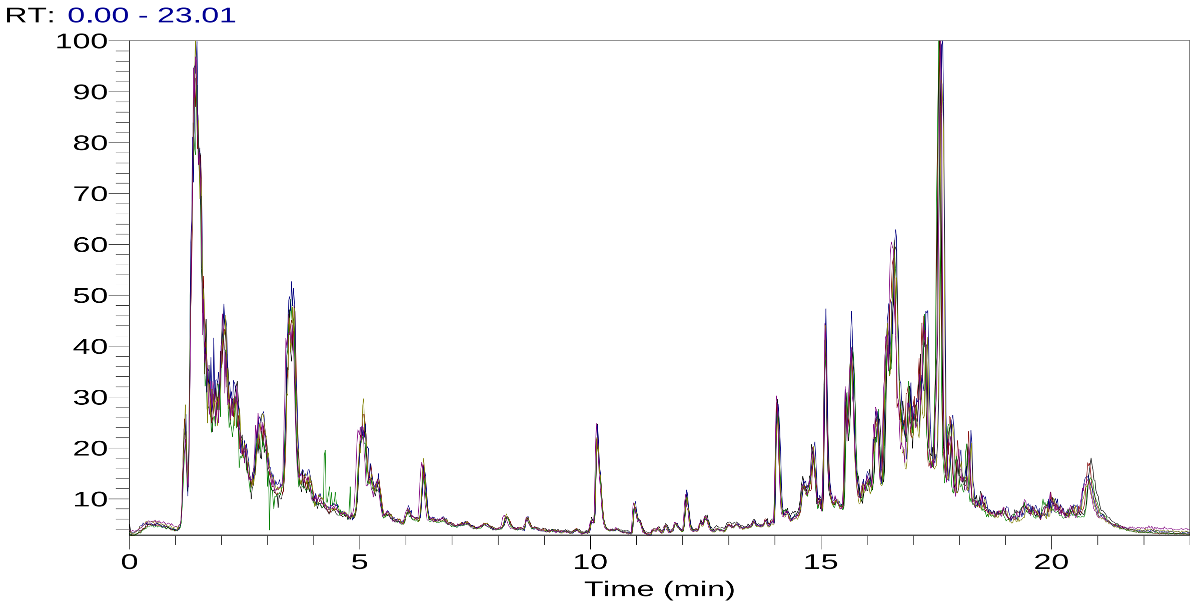

(B)

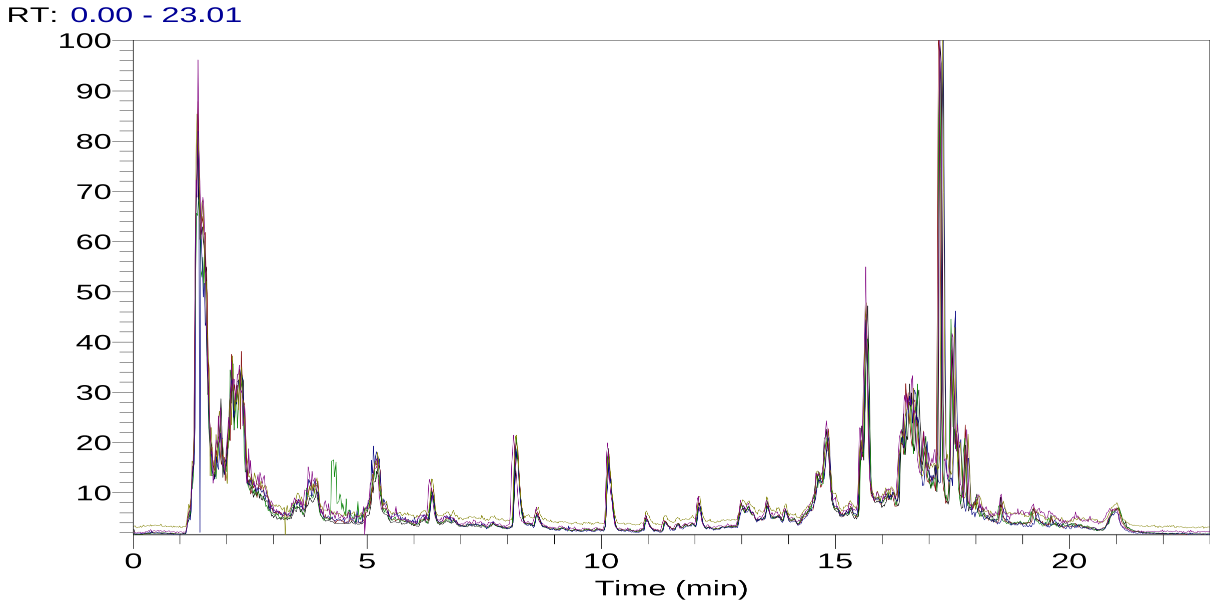

Fig. S1. Total ions chromatography (TIC) overlapping map of QC samples in the mass spectrometry results. (A) was detected in the positive ion mode; (B) was detected in the negative ion mode.

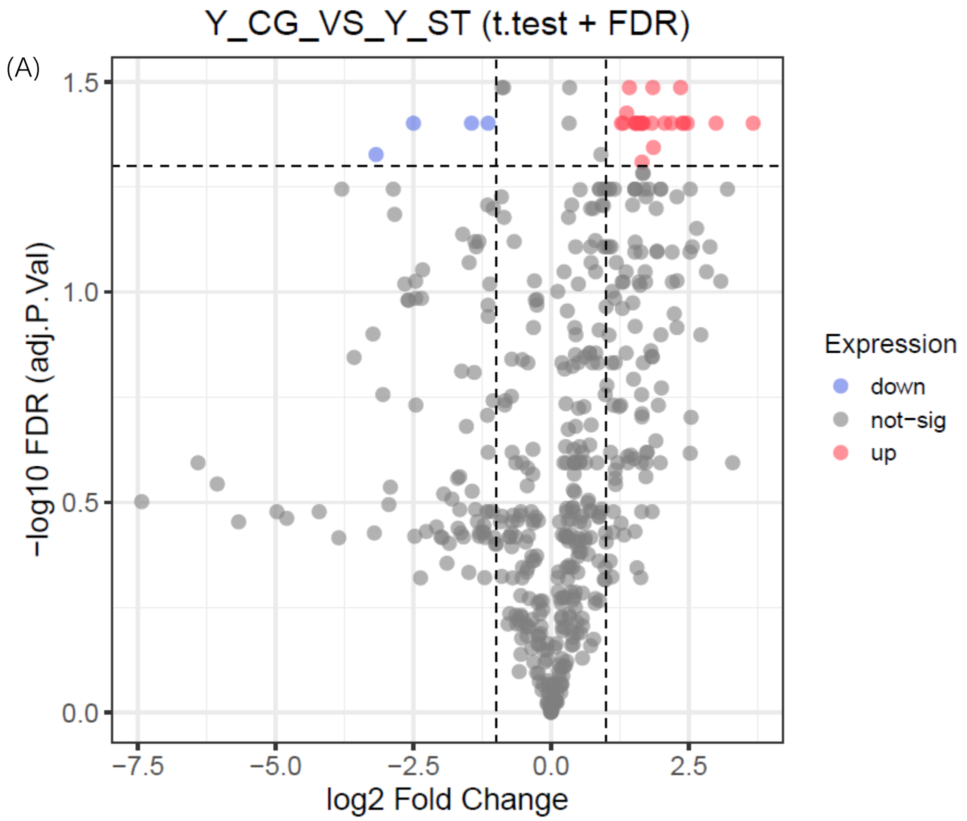
(A)
Y_CG_VS_Y_ST (t.test + FDR)
−log10 FDR (adj.P.Val)
1.5
1.0
0.5
0.0
−7.5
−5.0
−2.5
0.0
2.5
log2 Fold Change
Expression
down
not−sig
up

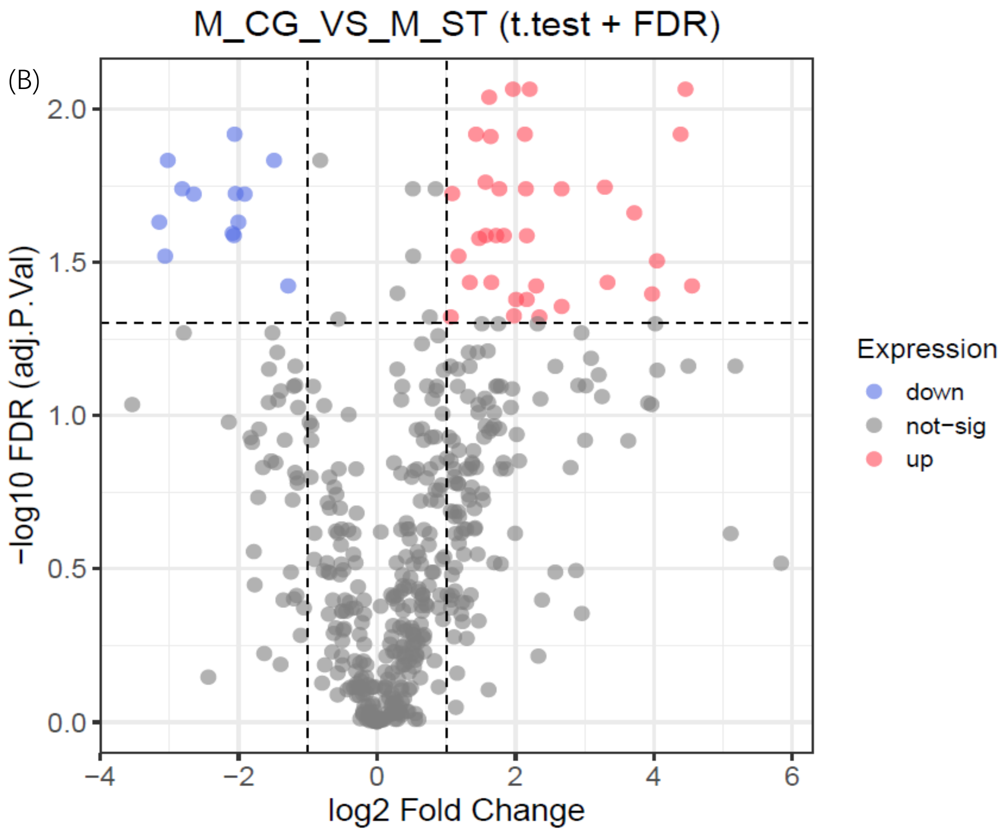
(B)
M_CG_VS_M_ST (t.test + FDR)
−log10 FDR (adj.P.Val)
2.0
1.5
1.0
0.5
0.0
−4
−2
0
2
4
6
log2 Fold Change
Expression
down
not−sig
up

(C)

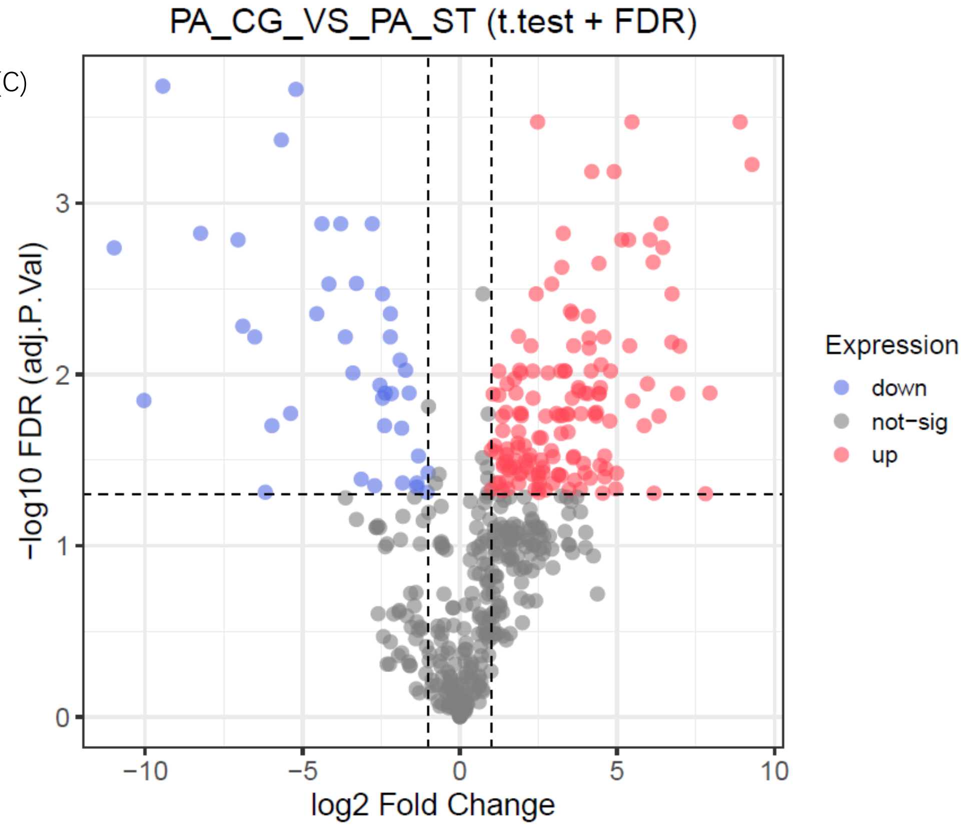
PA_CG_VS_PA_ST (t.test + FDR)
−log10 FDR (adj.P.Val)
log2 Fold Change
Expression
down
not−sig
up

(D)

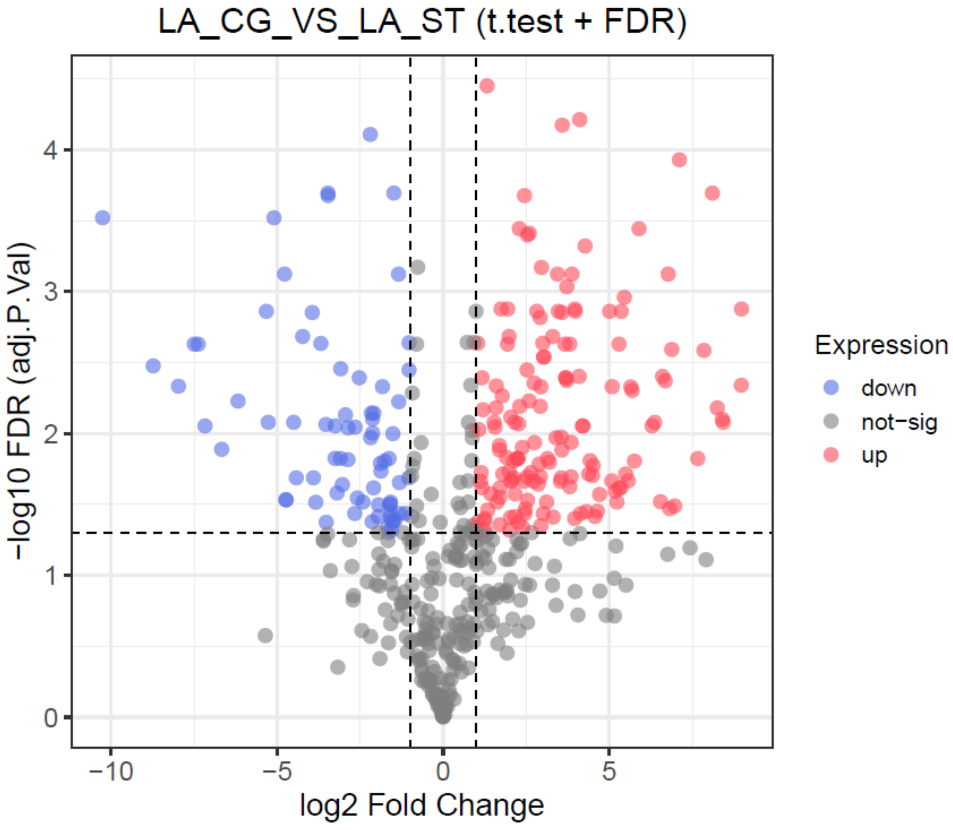
LA_CG_VS_LA_ST (t.test + FDR)
−log10 FDR (adj.P.Val)
log2 Fold Change
Expression
down
not−sig
up

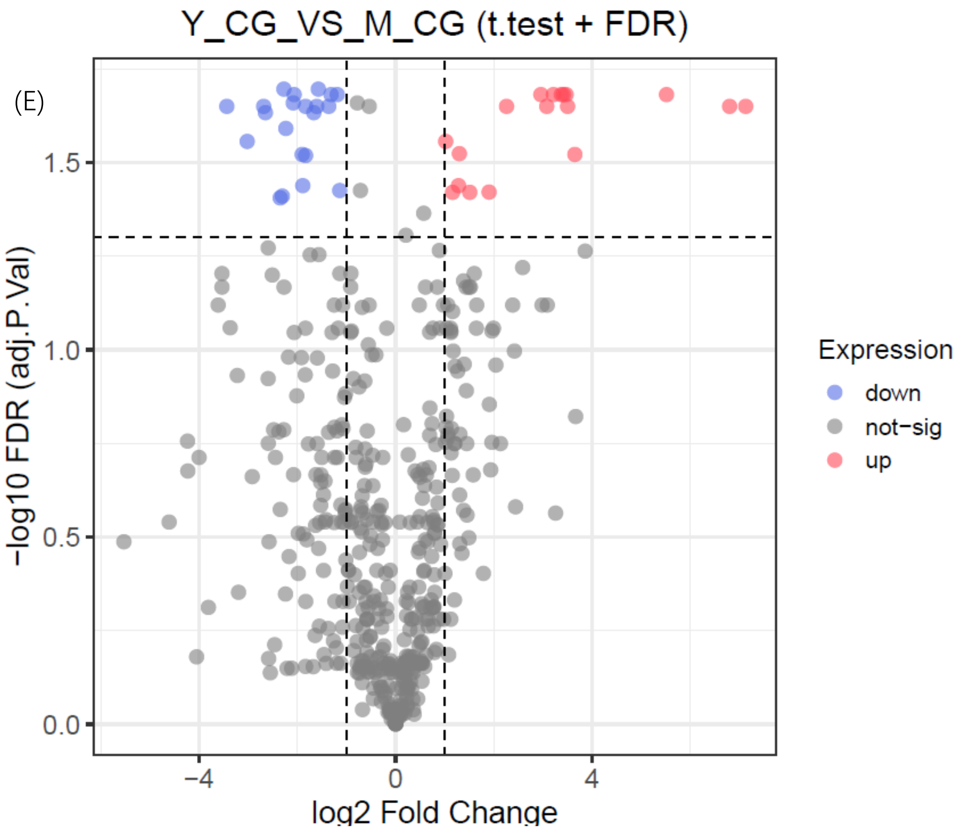
(E)
Y_CG_VS_M_CG (t.test + FDR)
−log10 FDR (adj.P.Val)
0.0
0.5
1.0
1.5
−4
0
4
log2 Fold Change
Expression
down
not−sig
up

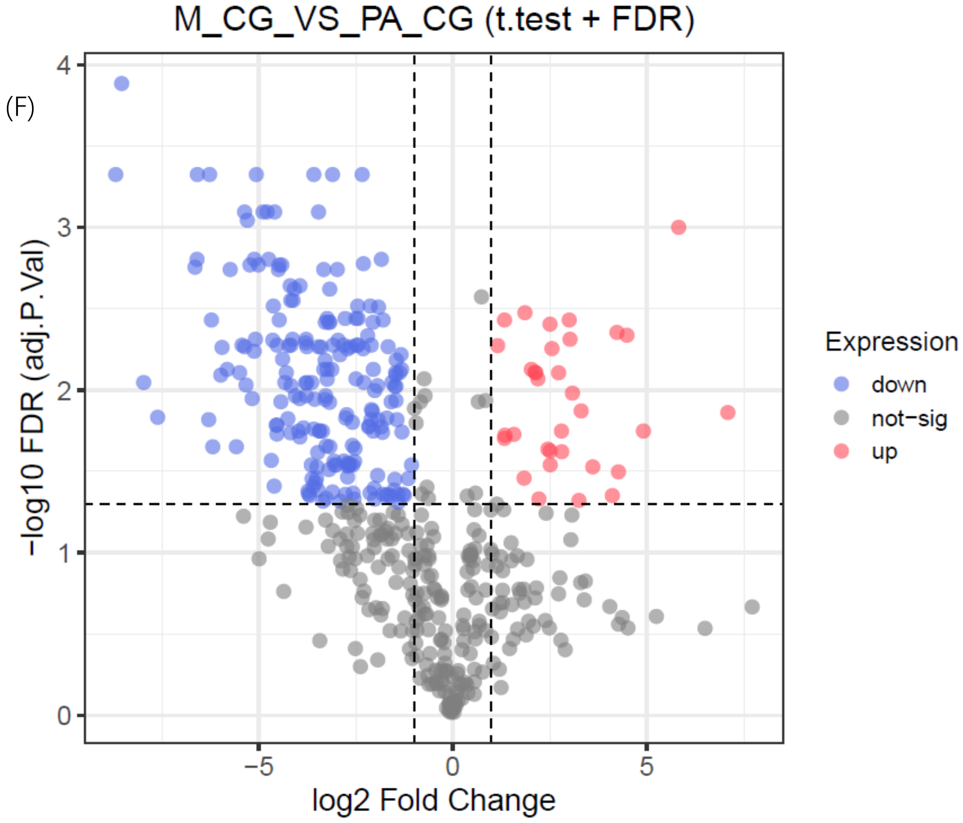
(F)
M_CG_VS_PA_CG (t.test + FDR)
−log10 FDR (adj.P.Val)
0
1
2
3
4
−5
0
5
log2 Fold Change
Expression
down
not−sig
up

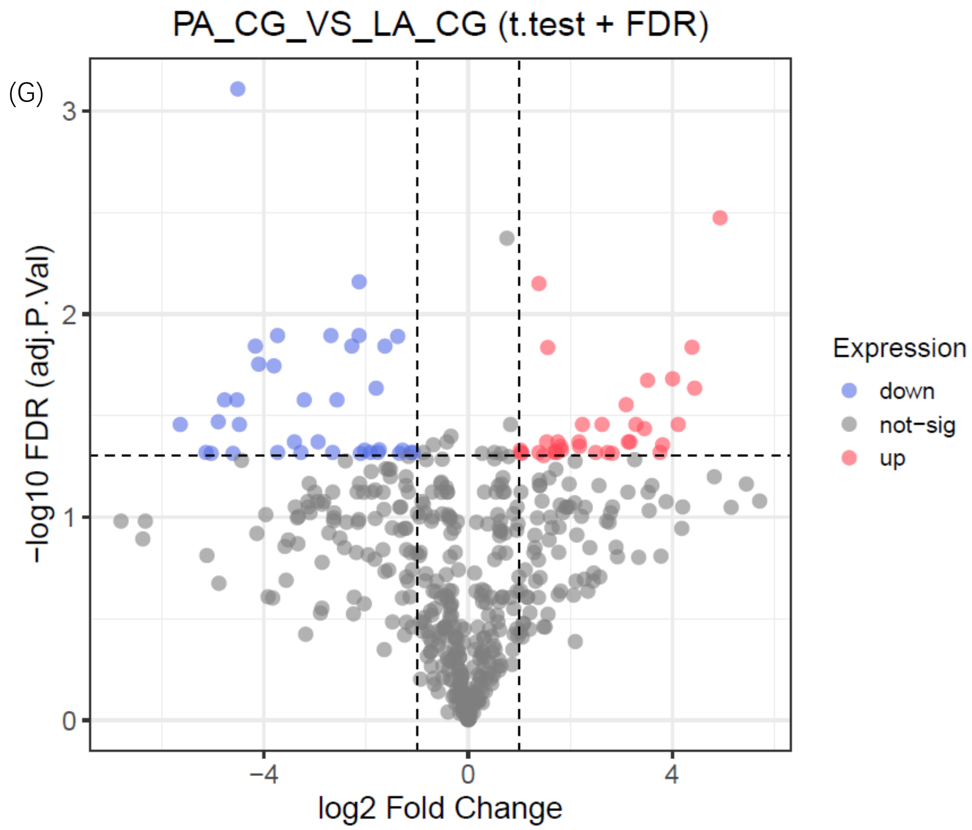
(G)
PA_CG_VS_LA_CG (t.test + FDR)
−log10 FDR (adj.P.Val)
3
2
1
0
−4
0
4
log2 Fold Change
Expression
down
not−sig
up

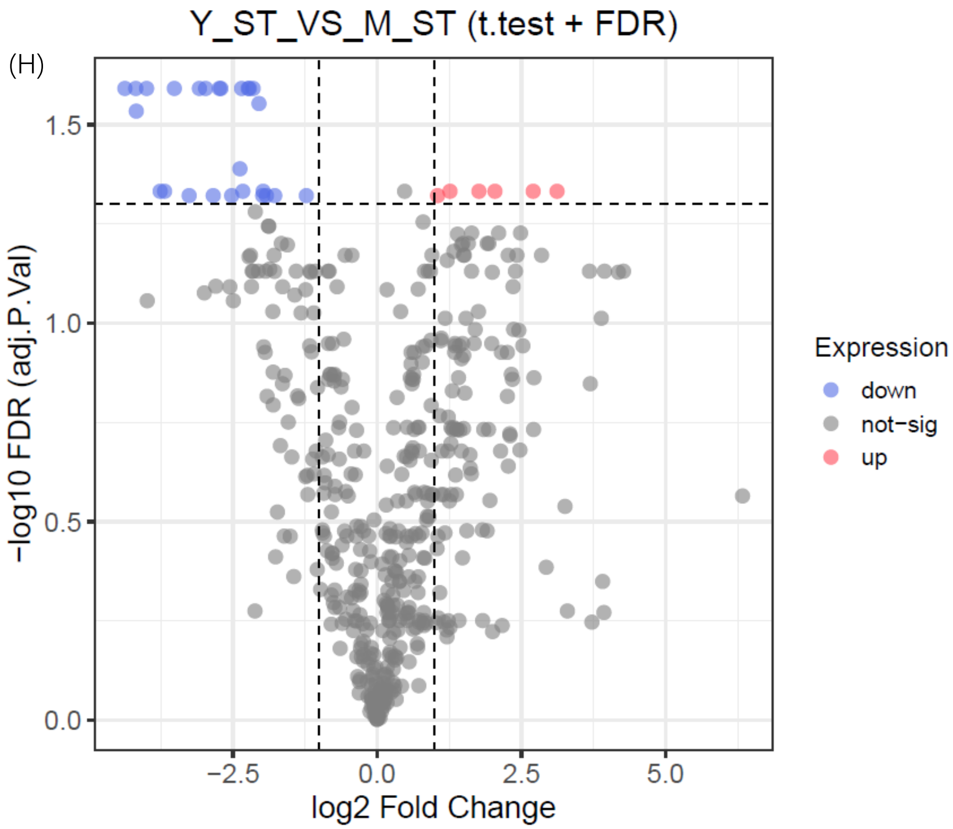
(H)
Y_ST_VS_M_ST (t.test + FDR)
−log10 FDR (adj.P.Val)
1.5
1.0
0.5
0.0
−2.5
0.0
2.5
5.0
log2 Fold Change
Expression
down
not−sig
up

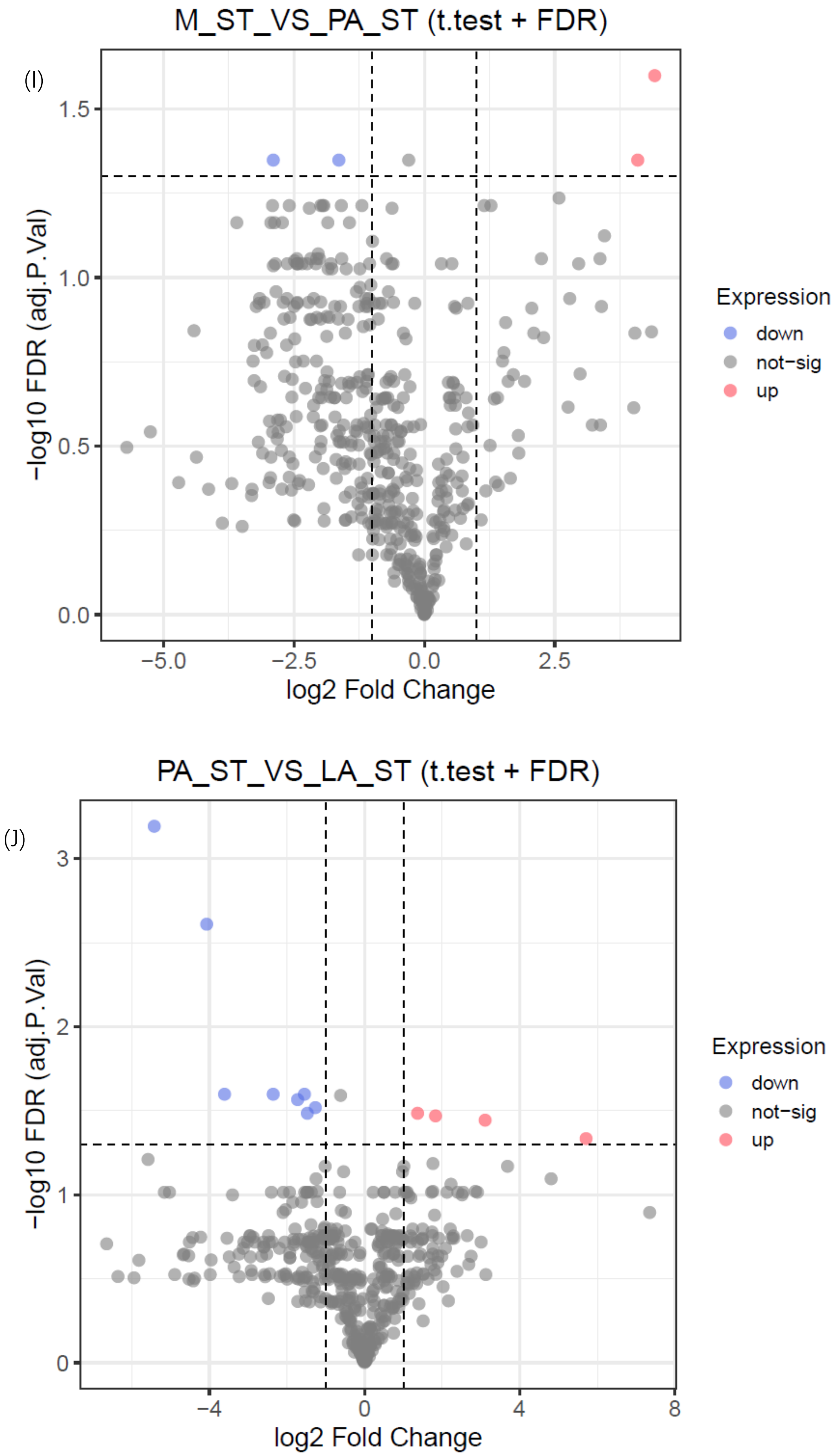
(I)
M_ST_VS_PA_ST (t.test + FDR)
−log10 FDR (adj.P.Val)
1.5
1.0
0.5
0.0
−5.0
−2.5
0.0
2.5
log2 Fold Change
Expression
down
not−sig
up
(J)
PA_ST_VS_LA_ST (t.test + FDR)
−log10 FDR (adj.P.Val)
3
2
1
0
−4
0
4
8
log2 Fold Change
Expression
down
not−sig
up

Fig. S2. Analysis of metabolites between CG and ST at the same stage and CG or ST between adjacent stages. Volcano plots. (A), Y-CG vs. Y-ST; (B), M-CG vs. M-ST; (C), PA-CG vs. PA-ST; (D), LA-CG vs. LA-ST; (E), Y-CG vs. M-CG; (F), M-CG vs. PA-CG; (G), PA-CG vs. LA-CG; (H), Y-ST vs. M-ST; (I), M-ST vs. PA-ST; (J), PA-ST vs. LA-ST.
